# *PRRT2* Mutations in Paroxysmal Kinesigenic Dyskinesia with Infantile Convulsions in a Taiwanese Cohort

**DOI:** 10.1371/journal.pone.0038543

**Published:** 2012-08-01

**Authors:** Yi-Chung Lee, Ming-Jen Lee, Hsiang-Yu Yu, Chien Chen, Chang-Hung Hsu, Kon-Ping Lin, Kwong-Kum Liao, Ming-Hong Chang, Yi-Chu Liao, Bing-Wen Soong

**Affiliations:** 1 Department of Neurology, Taipei Veterans General Hospital, Taipei, Taiwan; 2 Department of Neurology, National Yang-Ming University School of Medicine, Taipei, Taiwan; 3 Brain Research Center, National Yang-Ming University School of Medicine, Taipei, Taiwan; 4 Department of Neurology, National Taiwan University Hospital, Taipei, Taiwan; 5 Department of Neurology, Tri-Service General Hospital, Taipei, Taiwan; 6 Section of Neurology, Taichung Veterans General Hospital, Taichung, Taiwan; Oslo University Hospital, Norway

## Abstract

**Background:**

Mutations in the *PRRT2* gene have recently been identified in patients with familial paroxysmal kinesigenic dyskinesia with infantile convulsions (PKD/IC) and patients with sporadic PKD/IC from several ethnic groups. To extend these recent genetic reports, we investigated the frequency and identities of *PRRT2* mutations in a cohort of Taiwanese patients with PKD/IC.

**Methodology and Principal Findings:**

We screened all 3 coding exons of *PRRT2* for mutations in 28 Taiwanese patients with PKD/IC. Among them, 13 had familial PKD/IC and 15 were apparently sporadic cases. In total, 7 disparate mutations were identified in 13 patients, including 8 familial cases and 5 apparently sporadic cases. The mutations were not present in 500 healthy controls. Four mutations were novel. One patient had a missense mutation and all other patients carried *PRRT2* mutations putatively resulting in a protein truncation. Haplotype analysis revealed that 5 of the 7 patients with the *PRRT2* p.R217Pfs*8 mutation shared the same haplotype linked to the mutation.

**Conclusions and Significance:**

*PRRT2* mutations account for 61.5% (8 out of 13) of familial PKD/IC and 33.3% (5 out of 15) of apparently sporadic PKD/IC in the Taiwanese cohort. Most patients with the *PRRT2* p.R217Pfs*8 mutation in Taiwan likely descend from a single common ancestor. This study expands the spectrum of PKD/IC-associated *PRRT2* mutations, highlights the pathogenic role of *PRRT2* mutations in PKD/IC, and suggests genetic heterogeneity within idiopathic PKD.

## Introduction

Paroxysmal kinesigenic dyskinesia with infantile convulsions (PKD/IC) is characterized by childhood or adolescent onset recurrent transient unilateral or bilateral involuntary movements induced by sudden movements or startling, with or without a history of benign infantile convulsions. Attacks are usually less than 15 seconds in length, and manifest as dystonia, chorea, ballismus or a combination of these hyperkinesias. The frequency of attacks can range from more than 100 per day to less than one per month. Other characteristic features include preservation of consciousness and lack of pain during attacks, excellent control of attacks with carbamazepine or phenytoin, normal neurological examination and an age at onset usually between 1 and 20 years of age [1]. History of infantile convulsions is common. Most of idiopathic PKD patients have a family history, usually in an autosomal dominant mode [2].

The familial PKD locus was first mapped to the pericentromeric region of chromosome 16 in four French families with infantile convulsion and choreoathetosis (ICCA) syndrome [3], which was believed to be the same disorder as PKD/IC [4]. Soon after that, several pedigrees with pure PKD were also linked to the same area [5], [6]. Recently, mutations in the proline-rich transmembrane protein 2 gene (*PRRT2*) have been identified to cause PKD/IC [7]–[11]. The PRRT2 protein is highly expressed in the developing nervous system and localized to the axons, interacts with a synaptic protein, SNAP25, and may play a role in synaptic regulation [7], [10]. To further delineate the frequency and spectrum of *PRRT2* mutations, we screened a cohort of patients with PKD/IC in Taiwan.

**Figure 1:**
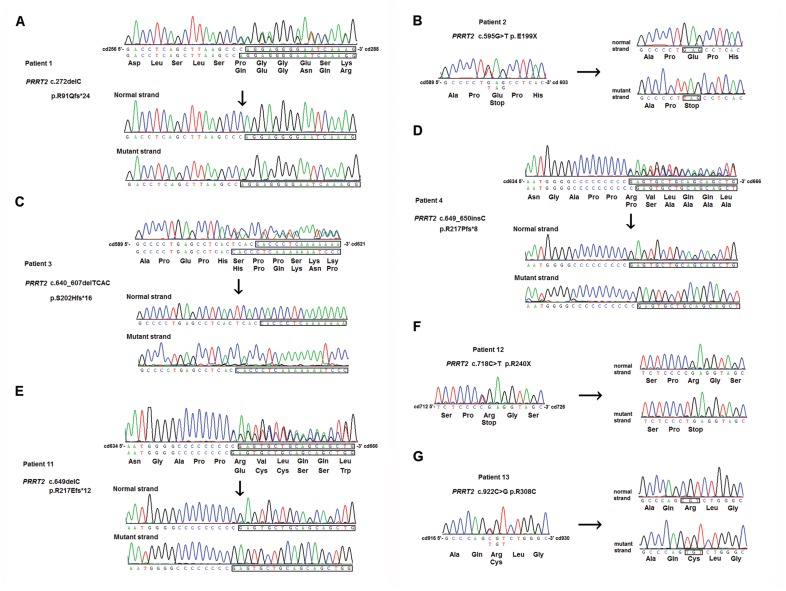
The *PRRT2* mutations identified in patients with paroxysmal kinesigenic dyskinesia with infantile convulsions in this study. The *PRRT2* heterozygous mutations, (A) c.272delC (p.P91Qfs*24), (B) c.595G>T (p.E199X), (C) c.604_607delTCAC (p.S202Hfs*16), (D) c.649_650insC (p.R217Pfs*8), (E) c.649del (p.R217Efs*12), (F) c.718C>T (p.R240X), and (G) c.922C>G (p.R308C) are shown by sequencing both the mutant and normal strands of the TA-subcloned PCR fragments.

## Methods

### Ethics Statement

The protocols for this study were approved by the Institutional Review Board of Taipei Veterans General Hospital. Written informed consent was obtained from all of the participants. For the patients younger than 20 years of age, the written informed contents were obtained from one of the parents and the patient simultaneously.

### Patients

Twenty-eight unrelated patients with idiopathic PKD/IC (25 males and 3 females; mean age, 23±6.8 years; mean age at onset, 12.4±4 years) were enrolled into this study. All the patients were of Han Chinese descent and were diagnosed with idiopathic PKD according to the clinical diagnostic criteria [1], which include identified kinesigenic triggers, short duration of attacks less than 1 minute, absence of consciousness impairment or pain during attacks, exclusion of other organic diseases and normal neurological examination. All the patients had a normal EEG and Brain CT or MRI examinations. Thirteen patients had a positive family history and fifteen patients were isolated cases. Family history was considered positive if the patient had at least 1 affected relative within 3 generations. One patient has been previously reported to have a *PRRT2* p.R240X mutation [10].

### Mutation Analysis

Genomic DNA was extracted from peripheral blood using standard protocols. Mutational analysis of the *PRRT2* gene was performed by PCR amplification and direct DNA sequencing. Primer sequences and PCR conditions are available in the supplemental method (Text S1). Both sense- and antisense-strands of all amplicons were sequenced using the Big Dye 3.1 di-deoxy terminator method (Applied Biosystems, Foster City, CA) and the ABI Prism 3700 Genetic Analyzer (Applied Biosystems). The amplicon sequences were compared against the published human gene sequence (*PRRT2*, NM_145239.2) in the National Center for Biotechnology Information (NCBI) database (http://www.ncbi.nlm.nih.gov) to identify potential mutations. After validation of the sequence variations in both sense and antisense strands, subcloning and subsequent sequencing of the amplicons were further performed to confirm the sequence changes. Sequence variants were tested in 500 unrelated healthy subjects of similar ethnic background using the same procedure of sequencing analyses.

### Bioinformatic Analysis

Phylogenetic conservation of the missense mutation was analyzed by aligning the amino acid sequences from several species (retrieved from the Entrez protein database in the NCBI database) using the ClustalX 2.012 program [12].

*In silico* prediction of the putative functional effects of the missense mutations was conducted with the PMut (http://mmb2.pcb.ub.es:8080/PMut/) [13], Polyphen2 [14] (http://tiddlyspace.com/bags/icgc_public/tiddlers/PolyPhen2) and SIFT (http://sift.jcvi.org) software [15].

**Figure 2:**
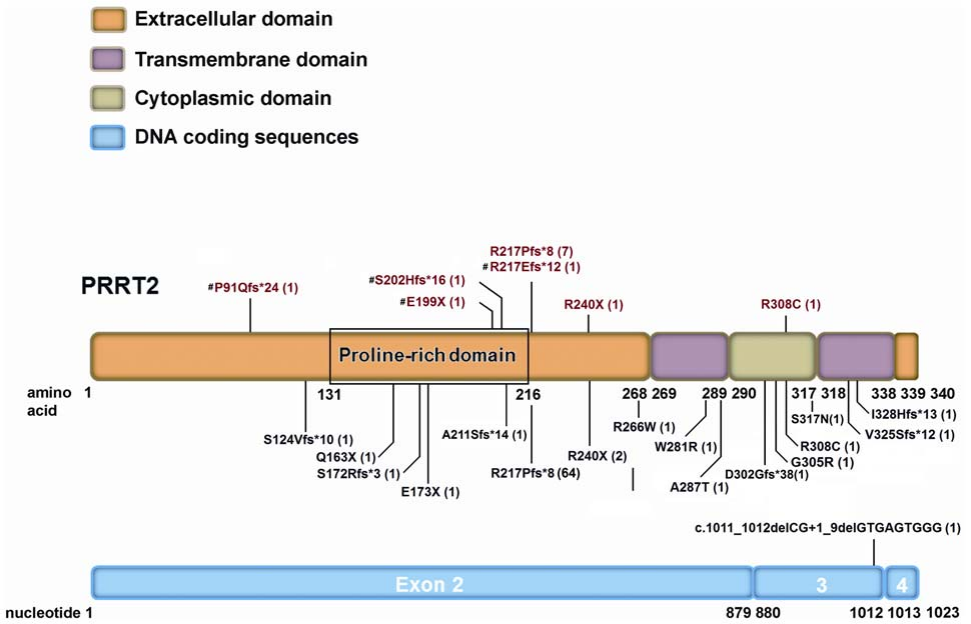
*PRRT2* mutations in patients with paroxysmal kinesigenic dyskinesia with infantile convulsions (PKD/IC). Twenty-one different mutations have been identified in *PRRT2* in familial and sporadic PKD/IC patients, with the majority resulting in a truncation of the PRRT2 protein. p.R217Efs*12 is particularly common and accounts for 75.5% (71/94) of the patients with *PRRT2* mutations. The mutations identified in this study are labeled in red and the novel ones with “**#**”, and those found in previous studies are labeled in black [7]–[11], [16]. Numbers within parentheses denote the number of the patients with the specific mutations.

### Haplotype Analyses of the Patients Carrying *PRRT2* p.R217Pfs*8 Mutation

Haplotype analyses were performed to explore the possible founder effect of p.R217Pfs*8 using 4 polymorphic microsatellite markers and 7 SNP markers flanking the *PRRT2* gene: D16S3068, D16S3100, D16S3022, rs9922666 and rs7205278 are telomeric, and rs4788186, rs7204252, rs889695, rs9926856, rs13332660, and D16S753 centromeric to *PRRT2*. These eleven markers cover a region of 5.71 Mb. All of the information regarding the primer sequences and allele marker sizes were obtained from the National Center for Biotechnology Information (NCBI) database (http://www.ncbi.nlm.nih.gov). The haplotypes were deduced by comparing the genotypes of each marker of multiple individuals within a pedigree.

**Table 1:** Genetic and clinical features of the patients with PKD/IC harboring *PRRT2* mutations.

Patient,gender	Age (y)	Age atonset (y)	Familial/AS	Nucleotidechanges	Amino acidchanges	Provokingfactors	Involuntarymovements	Durationof attacks	Frequencyof attacks	Currentmedication	Historyof IC
a1, M	24	11	familial	c.272delC	p.P91Qfs*24	SM	D/C	<20 sec	<1/m	CBZ	no
a2, F	44	10	familial	c.595G>T	p.E199X	SM/IM	C	<5 sec	<1/m	CBZ	no
a3, M	22	11	AS	c.604_607del TCAC	p.S202Hfs*16	SM	D	<10 sec	<1/w	no	no
4, M	21	13	familial	c.649_650insC	p.R217Pfs*8	SM/IM/S	D	<5 sec	<1/w	CBZ	no
5, M	15	7	familial	c.649_650insC	p.R217Pfs*8	SM/IM/S/s	D	<5 sec	<1/m	PHT	no
6, M	21	8	familial	c.649_650insC	p.R217Pfs*8	SM	D/C	<10 sec	<1/m	no	<4 y
7, M	18	8	familial	c.649_650insC	p.R217Pfs*8	SM	D	<10 sec	<1/d	CBZ	<2 y
8, M	22	20	AS	c.649_650insC	p.R217Pfs*8	SM/s	D/C	<1 min	<1/m	OXC	<4 y
9, M	27	11	AS	c.649_650insC	p.R217Pfs*8	SM/s	D	<20 sec	<1/m	no	no
10, M	21	10	AS	c.649_650insC	p.R217Pfs*8	SM	D	<10 sec	<1/w	no	no
a11, M	19	18	AS	c.649delC	p.R217Efs*12	SM	D/C	<10 sec	<1/day	CBZ	<4 y
12, M	21	15	familial	c.718C>T	p.R240X	SM	D	<20 sec	<5/day	CBZ	<2 y
13, M	19	10	familial	c.922C>G	p.R308C	SM	D/C	<5 sec	<5/day	no	no

Abbreviation: M  =  male; F  =  female; y  =  years; AS  =  apparently sporadic; SM  =  sudden movements; IM  =  intention to move; S  =  startle; s  =  stress; D  =  dystonia; C  =  choreoathetosis; CBZ  =  carbamazepine; m  =  month; w  =  week; PHT  =  phenytoin; OXC  =  oxcarbazepine; IC  =  infantile convulsions.

a These patients have novel mutations.

## Results

The sequencing of *PRRT2* gene in 28 unrelated patients with idiopathic PKD led to the identification of 4 truncating frameshift mutations, 2 nonsense mutations, and one missense mutation in 13 patients (46.4% of our cohort), including 8 out of the 13 familial cases (61.5%) and 5 out of the 15 apparently sporadic cases (33.3%). Among these mutations, p.R217Pfs*8 (c.649_650insC) was found in 7 patients (25% of our cohort), and p.P91Qfs*24 (c.272delC), p.E199X (c.595G>T), p.S202Hfs*16 (c.604_607delTCAC), p.R217Efs*12 (c.649delC), p.R240X (c.718C>T), and p.R308C (c.922C>G) in one single patient each (3.6%) (Figure 1 and Figure 2). None of these mutations were found in 500 control individuals in our population and the 1000 Genomes database (http://browser.1000genomes.org). The genetic and clinical features of the patients harboring these mutations are summarized in Table 1. There is no obvious difference in the clinical presentations of PKD between our familial patients with or without *PRRT2* mutations (Table S1). All three familial patients with histories of infantile convulsions carry *PRRT2* mutations (Table S1).

**Figure 3:**
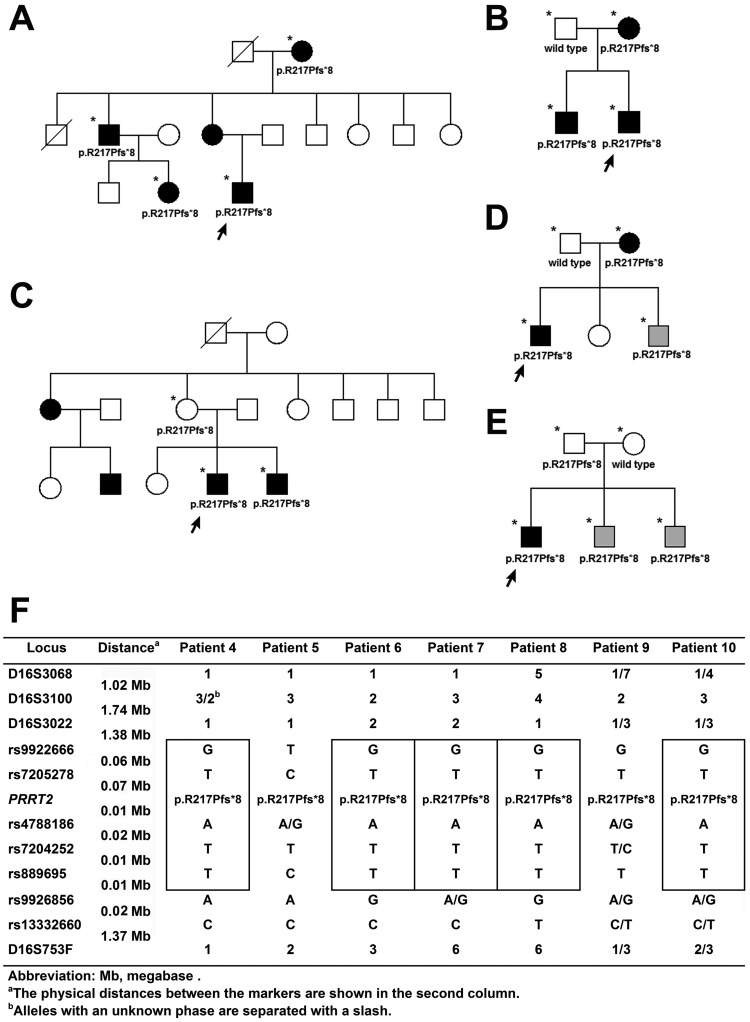
Haplotype analyses of the patients carrying *PRRT2* p.R217Pfs*8 mutation. Five unrelated PKD/IC pedigrees carry the *PRRT2* p.R217Pfs*8. Patients 4 (A), 5 (B), 6 (C), 7 (D), and 8 (E) are indicated with arrows. Asterisks (*) depict the individuals who were haplotyped. The squares and circles denote males and females, and the close and open symbols represent affected and unaffected members, respectively. The grey symbols denote undetermined disease status. The *PRRT2* genotype is labeled below the symbols. The alleles with an unknown phase are labeled and separated with a slash. The haplotypes linked to the *PRRT2* p.R217Pfs*8 in the seven unrelated index patients are showed in (F). Five patients shared a common haplotype at loci rs9922666, rs7205278, rs4788186, rs7204252, and rs889695 linked to the *PRRT2* p.R217Pfs*8 (G-T-p.R217Pfs*8-A-T-T).

Four of the identified mutations were novel, including p.P91Qfs*24, p.E199X, p.S202Hfs*16 and p.R217Efs*12. Notably, these 4 truncating frameshift or nonsense mutations have similar molecular features with most of the previously reported *PRRT2* mutations (Figure 2). The only one missense mutation, p.R308C, has been reported in a PKD patient previously [9]. The p.R308C mutation alters the highly conserved amino acid of the PRRT2 protein and was predicted to be deleterious by PMut, Polyphen-2, and SIFT.

Twenty-two individuals, including 14 patients, 5 carriers, and 3 unaffected individuals from 5 kindreds and 2 unrelated patients harboring the *PRRT2* p.R217Pfs*8 were haplotyped (Figure 3A-E). The haplotypes linked to the *PRRT2* p.R217Pfs*8 in the seven unrelated index patients were showed in Figure 3F. Five patients shared a common haplotype segment at loci rs9922666, rs7205278, rs4788186, rs7204252, and rs889695 linked to the *PRRT2* p.R217Pfs*8 (G-T-p.R217Pfs*8-A-T-T). Only patient 5 has a different haplotype (T-C-p.R217Pfs*8-A/G-T-C). No sample from the relatives of patients 9 and 10 was available for analyses,but patient 10 has a homozygous common haplotype (GTATT) at the five loci. Although the phase of the haplotype could not be deduced with certainty in patient 9, it is highly likely that he also has the common haplotype (G-T-p.R217Pfs*8-A-T-T).

## Discussion

Seventeen *PRRT2* mutations have recently been identified in patients with familial or apparently sporadic PKD/IC from several ethnic groups [7]–[11], [16]. A vast majority of the mutations (11/17) were premature termination or frameshift mutations resulting in a truncation of the PRRT protein (Figure 2). The rest were missense mutations. Interestingly, p.R217P*8 was found in approximately 80% of PKD patients with *PRRT2* mutations [7]–[11], [16]. All identified families displayed an autosomally dominant inheritance.

In our cohort of 28 unrelated patients with idiopathic PKD/IC, 7 *PRRT2* mutations were identified from 13 patients. Similar to the recent reports, the great majority of them were truncating mutations (6 out of 7, 85.7%). The p.R217P*8 accounts for 53.8% (7/13) of PKD *PRRT2* mutations in our population. The only one missense mutation, p.R308C, had earlier been identified in a patient with sporadic PKD [9]. Although we had difficulty recruiting family members of the patient with *PRRT2* p.R308C to study the genotype-phenotype co-segregation, the pathogenic role of p.R308C is still strongly implicated by the involvement of a highly conserved amino acid, deleterious nature predicted by three popular bioinformatics tools, and its absence in 1000 control chromosomes.

The four novel mutations identified in this study all putatively result in a truncation of the PRRT2 protein and are located in the N-terminal extracellular domain (Figure 2), leading to a loss of other parts of the PRRT2 protein. The function of PRRT2 protein is still poorly characterized. Recent *in vivo* and *in vitro* studies demonstrated that PRRT2 is highly expressed in the developing nervous system and localized on the cell membrane, predominantly in the axons [7], [10]. It is not surprising that the truncating mutations may lead to a significant reduction of protein expression and also a loss of the transmembrane property, which conceivably impairs the function of PRRT2 protein [7], [10].

The observed frequency of mutations in our PKD cohort is 61.5% (8/13) in familial cases and 33.3% (5/15) in apparently sporadic cases, which are a bit smaller than those observed in one previous study of *PRRT2* mutations from Beijing, China (75% (3/4) in familial cases and 34.5% (10/29) in apparently sporadic cases) [9]. Absence of *PRRT2* mutations in PKD families was also found in two previous reports [7], [9]. It is likely that genetic heterogeneity does exist within idiopathic PKD, and therefore, mutations in other genes are awaiting to be identified.

Seven unrelated PKD patients with *PRRT2* mutations in our cohort have p.R217P*8 and five of them share a common haplotype segment linked to the mutation (Figure 3), indicating the existence of a major founder for *PRRT2* p.R217P*8, which actually might not be surprising in PKD given its mild impact on daily activities and fertility. The common haplotype linked to the *PRRT2* p.R217P*8 in our cohort is different from the three haplotypes reported in the patients with the same mutations from Southeast China [7]. Given the geographical proximity between Taiwan and Southeast China, many genetic diseases may have common founders. The discrepancy of the founder haplotypes suggests that *PRRT2* p.R217P*8 is liable to occur spontaneously. *PRRT2* p.R217P*8 (c.649_650insC) arises from an insertion of one “C” after a 9 consecutive single nucleotide “C” repeat (Figure 1D), which is prone to occur by a slipped-strand mispairing mechanism during DNA replication. Two carriers were found during the haplotype studies (Figure 3C, E), suggesting that the incomplete penetrance of the *PRRT2* mutations is likely to occur. Although the carriers all denied any histories relevant to PKD or infantile convulsions, some of them may be in denial or frankly denying past symptoms because of the guilt they feel for having ‘transmitted PKD/IC’ to their children.

In conclusion, this study demonstrated the variety and frequency of *PRRT2* mutations in a Taiwanese cohort with idiopathic PKD. Our findings expanded the spectrum of *PRRT2* mutations, suggested genetic heterogeneity, and further highlighted the importance of mutations in *PRRT2* in the pathogenesis of PKD.

## Supporting Information

Table S1 **Clinical features of the index patients with familial PKD/IC with or without PRRT2 mutations.**
(DOCX)

Text S1 **Primers sequences and PCR conditions for mutational analyses of** ***PRRT2.***
(DOC)
