# Supplementary material for: PRRT2 Mutations in Paroxysmal Kinesigenic Dyskinesia with Infantile Convulsions in a Taiwanese Cohort
Source: PLoS One. 2012 Aug 1;7(8):e38543. doi: 10.1371/journal.pone.0038543 (PMC3409860; doi:10.1371/journal.pone.0038543)
Supplement: Table S1 — Clinical features of the index patients with familial PKD/IC with or without PRRT2 mutations. (DOCX) [file pone.0038543.s001.docx]

**Supplementary table 1.** Clinical features of the index patients with familial PKD/IC with or without *PRRT2* mutations

| Patient, gender | Age (y) | Age at onset (y) | Familial | Nucleotide changes | Amino acid changes | Provoking factors | Involuntary movements | Duration of attacks | Frequency of attacks | Current medication | History of IC |
| --- | --- | --- | --- | --- | --- | --- | --- | --- | --- | --- | --- |
| *PRRT2* mutation positive | | | | | | | | | | | |
| M | 24 | 11 | familial | c.272delC | p.P91Qfs*24 | SM | D/C | <20 sec | <1/m | CBZ | no |
| F | 44 | 10 | familial | c.595G>T | p.E199X | SM/IM | C | <5 sec | <1/m | CBZ | no |
| M | 21 | 13 | familial | c.649_650insC | p.R217Pfs*8 | SM/IM/S | D | <5 sec | <1/w | CBZ | no |
| M | 15 | 7 | familial | c.649_650insC | p.R217Pfs*8 | SM/IM/S/s | D | <5 sec | <1/m | PHT | no |
| M | 21 | 8 | familial | c.649_650insC | p.R217Pfs*8 | SM | D/C | <10 sec | <1/m | no | <4 y |
| M | 18 | 8 | familial | c.649_650insC | p.R217Pfs*8 | SM | D | <10 sec | <1/d | CBZ | < 2 y |
| M | 21 | 15 | familial | c.718C>T | p.R240X | SM | D | <20 sec | <5/day | CBZ | <2 y |
| M | 19 | 10 | familial | c.922C>G | p.R308C | SM | D/C | <5 sec | <5/day | no | no |
| *PRRT2* mutation negative | | | | | | | | | | | |
| F | 26 | 16 | familial |  |  | SM | D/C | <10 sec | <1/m | CBZ | no |
| M | 22 | 12 | familial |  |  | SM | D | <5 sec | <1/day | CBZ | no |
| M | 24 | 11 | familial |  |  | SM/S | D | <10 sec | <1/m | CBZ | no |
| M | 20 | 17 | familial |  |  | SM/s | D | <10 sec | <1/m | OXC | no |
| M | 19 | 11 | familial |  |  | SM/s | D/C | <10 sec | <1/m | CBZ | no |

Abbreviation: M = male; F = female; y = years; AS = apparently sporadic; SM = sudden movements; IM = intention to move; S = startle; s = stress; D = dystonia; C = choreoathetosis; CBZ = carbamazepine; m = month; w = week; PHT = phenytoin; OXC = oxcarbazepine; IC = infantile convulsions.
