# Supplementary material for: PRRT2 Mutations in Paroxysmal Kinesigenic Dyskinesia with Infantile Convulsions in a Taiwanese Cohort
Source: PLoS One. 2012 Aug 1;7(8):e38543. doi: 10.1371/journal.pone.0038543 (PMC3409860; doi:10.1371/journal.pone.0038543)
Supplement: Text S1 — Primers sequences and PCR conditions for mutational analyses of PRRT2. (DOC) [file pone.0038543.s002.doc]

# Supplemental Materials

Primers sequences and PCR conditions for mutational analyses of *PRRT2*

Forward primer Reverse primer

PPRT2-Exon2A 5'-ctcctcctcttccagggttt-3' 5'-tttttgagggtggtgagtga-3'

PRRT2-Exon2B 5'-tctgagagtgtaggggaaaagc-3' 5'-ctagggagaggcaaacaaagg-3'

PRRT2-Exon34 5'-tccacctgatcccttctgg-3' 5'-caggctcccttggtccttag-3'

PCR was performed in solution with a final volume of 10 ul, containing 0.5 uM of each primer, 200 uM of dNTP (each), 10X buffer, 0.25 unit of Tag polymerase (TAKARA), and 20 ng genomic DNA. The following PCR conditions were employed: 95°C for 5 min; 30 cycles at 95°C for 30 s, 60°C for 30 s, and 72°C for 1 min; 72°C for 10 min, then maintained at 4°C.
